# Regulatory Potential of Long Non-Coding RNAs (lncRNAs) in Boar Spermatozoa with Good and Poor Freezability

**DOI:** 10.3390/life10110300

**Published:** 2020-11-21

**Authors:** Leyland Fraser, Łukasz Paukszto, Anna Mańkowska, Paweł Brym, Przemysław Gilun, Jan P. Jastrzębski, Chandra S. Pareek, Dibyendu Kumar, Mariusz Pierzchała

**Affiliations:** 1 Department of Animal Biochemistry and Biotechnology, Faculty of Animal Bioengineering, University of Warmia and Mazury in Olsztyn, 10-719 Olsztyn, Poland; anna.mankowska@uwm.edu.pl; 2 Department of Plant Physiology, Genetics and Biotechnology, Faculty of Biology and Biotechnology, University of Warmia and Mazury in Olsztyn, 10-719 Olsztyn, Poland; lukasz.paukszto@uwm.edu.pl (Ł.P.); jan.jastrzebski@uwm.edu.pl (J.P.J.); 3 Department of Animal Genetics, Faculty of Animal Bioengineering, University of Warmia and Mazury in Olsztyn, 10-719 Olsztyn, Poland; pawbrym@uwm.edu.pl; 4 Department of Local Physiological Regulations, Institute of Animal Reproduction and Food Research of the Polish Academy of Sciences, Bydgoska 7, 10-243 Olsztyn, Poland; p.gilun@pan.olsztyn.pl; 5 Institute of Veterinary Medicine, Faculty of Biological and Veterinary Sciences, Nicolaus Copernicus, University, 87-100 Toruń, Poland; pareekcs@umk.pl; 6 Waksman Institute of Microbiology, Rutgers, The State University of New Jersey, Piscataway, NJ 08554, USA; dk@waksman.rutgers.edu; 7 Institute of Genetics and Animal Breeding, Polish Academy of Sciences, Jastrzębiec, 05-552 Magdalenka, Poland; m.pierzchala@ighz.pl

**Keywords:** boar, semen freezability, transcriptome assembly, lncRNAs, target genes

## Abstract

Long non-coding RNAs (lncRNAs) are suggested to play an important role in the sperm biological processes. We performed *de novo* transcriptome assembly to characterize lncRNAs in spermatozoa, and to investigate the role of the potential target genes of the differentially expressed lncRNAs (DElncRNAs) in sperm freezability. We detected approximately 4007 DElncRNAs, which were differentially expressed in spermatozoa from boars classified as having good and poor semen freezability (GSF and PSF, respectively). Most of the DElncRNAs were upregulated in boars of the PSF group and appeared to significantly affect the sperm’s response to the cryopreservation conditions. Furthermore, we predicted that the potential target genes were regulated by DElncRNAs in *cis* or *trans*. It was found that DElncRNAs of both freezability groups had potential *cis*- and *trans*-regulatory effects on different protein-coding genes, such as *COX7A2L*, *TXNDC8* and *SOX-7*. Gene Ontology (GO) enrichment revealed that the DElncRNA target genes are associated with numerous biological processes, including signal transduction, response to stress, cell death (apoptosis), motility and embryo development. Significant differences in the *de novo* assembled transcriptome expression profiles of the DElncRNAs between the freezability groups were confirmed by quantitative real-time PCR analysis. This study reveals the potential effects of protein-coding genes of DElncRNAs on sperm functions, which could contribute to further research on their relevance in semen freezability.

## 1. Introduction

Spermatozoa comprise a wide repertoire of RNAs that are implicated in several biological processes associated with spermatogenesis, fertilization and embryo development [1,2,3,4,5]. In eukaryotes, the vast majority of gene transcripts are non-coding RNAs (ncRNAs) [6]. While once thought to be “junk” in the sequences of genomes, accumulating evidence has shown that ncRNAs are implicated in numerous important biological processes, such as transcription, splicing, translation, epigenetic modifications, cell development and differentiation [3,5,6,7,8]. Recently, several reports have focused on the biological functions of long non-coding RNAs (lncRNAs) [4,6,8]. Evidence has shown that the lncRNAs represent the largest part of the transcriptome in mammals, but only a small proportion has been functionally characterized [7]. The lncRNAs are classified as regulatory molecules, which are defined as transcripts with a length greater than 200 nucleotides, and most of them exhibit cell-type specific expression [4,5,8]. Most lncRNAs can be broadly classified into four groups based on their genomic locations: sense lncRNAs, antisense lncRNAs, intronic lncRNA and long intergenic non-coding RNAs (lincRNAs) [4,7].

It is noteworthy that ncRNAs (including lncRNAs), which have no discernable coding potential, are implicated in the regulation of gene expression and affect spermatogenesis through co-transcriptional mechanisms [1,2,4,9,10]. It has been suggested that there is a mechanism by which lncRNAs could affect sperm maturation and post-fertilization [9]. Accordingly, lncRNAs could modulate protein activity by binding to target proteins [5]. Evidence has shown that the lncRNAs in spermatozoa are involved in processes related to apoptosis, heat shock response and cryo-damage [9,10,11]. Moreover, the function of lncRNAs in spermatozoa is predicted by assessing their potential *cis*- or *trans*-regulatory effects, depending on whether they exert their function on the target genes on the same allele from which they are transcribed [1,9,10,11]. The expression patterns of lncRNAs are correlated with neighboring mRNAs, suggesting that lncRNAs might have a regulatory role in sperm functions [10]. Accumulating evidence has indicated that the differential expression patterns of lncRNAs in spermatozoa could affect the regulation of the corresponding target mRNAs, thereby affecting sperm functions, such as motility [1,4,10].

Presently, very few studies have investigated the roles of differentially expressed lncRNAs (DElncRNAs) in sperm functions. As regards boar spermatozoa, a previous study has reported on the role of lncRNAs in sperm functions [12]. It should be emphasized that our understanding of the roles of lncRNAs in sperm functions and cryo-damage is still limited. Recently, it has been shown that variations in the profiles of differentially expressed genes are associated with the cryo-tolerance of boar spermatozoa [13,14]. We hypothesized that the expression profiles of DElncRNAs might also be associated with the freezability of boar semen. We performed *de novo* transcriptome assembly of spermatozoa from the Polish large white (PLW) boars to identify and characterize lncRNAs, and to assess the roles of potential target genes of the DElncRNAs in terms of sperm freezability.

## 2. Materials and Methods

### 2.1. Chemical and Media

All chemicals were bought from Sigma Chemical Company (St. Louis, MO, USA), unless otherwise stated. The fluorescent probes, 5,5′,6,6′-tetrachloro-1,1′,3,3′-tetraethylbenzimidazolylcarbocyanine iodide (JC-1), SYBR-14 (Live/Dead Sperm Viability Kit) and propidium iodide (PI) were purchased from Molecular Probes (Eugene, OR, USA).

### 2.2. Animal and Semen Collections

In this study, nine boars (average age of 2 years) were used and approximately 5 to 7 sperm-rich fractions (SRFs) were collected from each animal. Sperm samples that had a minimum of 70% total motility (TMOT) and at least 85% spermatozoa with normal morphology were used in the study. Animal experiments were carried out in accordance with the guidelines set out by the Local Ethics Committee. Approval of the Local Ethics Committee for experiments on boars (semen collection procedure) has not been required since 15/01/2015.

### 2.3. Semen Processing Procedure and Quality Assessment

#### 2.3.1. Semen Cryopreservation

The SRFs were frozen according to a cryopreservation protocol [15,16], with some modifications [17]. Following the centrifugation of the extended semen, the sperm pellets were re-suspended in an extender containing 11% lactose (*w*/*v*) and lipoprotein fractions of ostrich egg yolk (LPFo) extender. The LPFo-extended semen was cooled (5 °C for 2 h), diluted (2:1) and packaged into sterilized aluminum tubes, before being loaded onto a programmable computer freezing machine (Ice Cube 1810, SY-LAB, Purkersdorf, Austria). Frozen sperm samples were kept in liquid nitrogen before post-thaw (PT) assessment.

#### 2.3.2. Motility Parameters Analyzed by the Computer-Assisted Sperm Analysis (CASA) System

The Computer-Assisted Sperm Analysis (CASA) system (HTR-IVOS 12.3, Hamilton Thorne Biosciences, Beverly, MA, USA) was used to monitor sperm motility and velocity parameters [14]. The software settings used for the CASA system were those recommended by the manufacturer for analysis of boar spermatozoa. The CASA system measured the total motility (TMOT, %), progressive motility (PMOT, %), velocity straight line (VSL, µm/sec) and the velocity average path (VAP, µm/sec).

#### 2.3.3. Membrane Integrity Assessment

Sperm membrane integrity was represented by mitochondrial membrane potential (MMP), plasma membrane integrity (PMI), normal apical ridge (NAR) acrosome integrity (intact acrosome) and DNA fragmentation.

The percentages of spermatozoa with MMP were evaluated with the fluorescent lipophilic cation JC-1 and PI fluorescent dyes (Molecular Probes, Eugene, OR, USA) [18,19]. Slides stained with JC-1/PI dyes were examined at ×600 magnification under a fluorescence microscope (Olympus CH 30, Tokyo, Japan). Spermatozoa exhibiting only orange-red fluorescence were considered as viable cells with functional mitochondria. A minimum of 100 spermatozoa were counted per slide, and two slides were evaluated per sample.

Sperm PMI was monitored with the SYBR-14 and PI fluorescent probes, using the Live/Dead Sperm Viability Kit (Molecular Probes, Eugene, OR, USA) [20]. A minimum of 100 cells per slide were examined at × 600 magnification under a fluorescence microscope (Olympus CH 30), and two slides were evaluated per sample.

The percentage of spermatozoa with normal apical ridge (NAR) acrosome integrity was assessed, according to a previously described method [21,22]. A minimum of 100 cells per slide, two slides per sample, were examined under a bright light microscope, equipped with oil-immersion lens, at ×1000 magnification (Olympus BX 41, Olympus, Tokyo, Japan), and was considered as a sperm with NAR acrosome integrity or damaged apical ridge acrosome.

The Comet assay was used to measure sperm DNA fragmentation [15,16]. The sperm samples, stained with ethidium bromide (Sigma Chemical Company, St. Louis, MO, USA), were assessed at ×400 magnification under a fluorescence microscope (Olympus BX 41, Tokyo, Japan). Spermatozoa were classified as non-fragmented DNA and fragmented DNA cells, and slides were analyzed in duplicate.

### 2.4. De Novo Transcriptome Assembly

The RNA-Seq datasets that were submitted to the National Center for Biotechnology Information (NCBI) Sequence Read Archive (SRA) database (BioProject: PRJNA415904; accession number, SRP121647; BioProject: PRJNA454080, accession number: SRP143583) were used for transcriptome assembly. The datasets represent the RNA-Seq from nine PLW boars, four boars were classified as having good semen freezability, GSF (G01, G04, G09 and G17) and five boars were classified as having poor semen freezability, PSF (P30, P36, P37, P38 and P39). Transcriptomes analysis of spermatozoa from six boars (G01, G09, G17, P30, P38 and P39) has been published in our recent study [14]. We used a similar transcriptome sequencing procedure for the other three boars, G04, P36 and P37 [14].

For transcriptome assembly, the cDNA libraries were prepared as described in our recent study [14]. The sequencing was performed on Illumina NextSeq500 instrument, and the quality control of the paired-end (2 × 75 bp) raw sequences was checked with FastQC software (v0.11.4) (Babraham Institute, Cambridge, UK). Paired-end reads with a length shorter than 35 bp were removed from downstream analysis using Trimmomatic tool v. 0.32 [23]. Trimmed RNA-Seq was *de novo* assembled using the Trinity software v.2.6.6 [24], with a 64-core processor and a 120 GB RAM server (Regional Information Technology Center, Olsztyn, Poland). A minimum transcript length of 200 nucleotides (nt) was included in the assembled contigs of the spermatozoa transcriptome. Statistics of de novo transcriptome assembly were performed with the Trinitystats.pl script, and included average contig length, GC (guanine-cytosine) content, total assembled bases and N50 parameters. All contigs were annotated using Swiss-Prot tools protein database [25] and Blastx algorithm with E-value threshold of 1E-5 [26]. Contigs were filtered on the basis of their coding sequences, using TransDecoder (http://transdecoder.github.io) to identify putative ORFs (open reading frames) in each transcript. The selected sequences were blasted against Swis-Prot protein database, using Blastp algorithm with E-value threshold of 1E-5. Conserved domains, signal peptides and transmembrane regions the selected sequences were removed, using available software packages: HMMER and PFAM tools [27]; and with the SignalP and TmHMM tools available at the database (https://services.healthtech.dtu.dk). Trinotate parsed all the results in the SQlite local database and created tab-delimited annotation file.

### 2.5. Identification of DElncRNAs

We used the RNA-Seq by Expectation Maximization (RSEM) tool to measure the expression values of the significantly differentially expressed lncRNA transcripts (DElncRNAs) [28]. The read count matrix was used as the input for the normalization procedure based on the trimmed means of M-values (TMM) of the count matrix [29]. The expression levels of the transcripts were normalized, using the fragments per kilobase of transcripts per million reads mapped (FPKM) method. Statistical analysis was performed with DESeq2 Bioconductor R package to identify the DElncRNA transcripts between the GSF and PSF groups [29]. Differences in the transcript expression between the GSF and PSF groups were evaluated, using the *p*-adjusted values (false discovery rate, FDR) less than 0.05 (FDR < 0.05) and log2 fold-change (log2FC), with values more than 1 (>1) and less than −1 (<−1) as the threshold. The predictor tools used to screen the coding potential of the lncRNAs were as follows: coding potential calculator, CPC (score < −1) [30], and predictor of long non-coding RNAs and messenger RNAs based on an improved k-mer scheme (PLEK) (score < 0) [31]. Transcripts without coding potential were also blasted against the Rfam database (Blast2GO, v.5.2.5) to remove all housekeeping RNAs, such as tRNA, rRNA, snRNA and snoRNA [32]. Transcripts revealing coding potential were filtered out and the remaining transcripts were considered as potential candidate lncRNAs. The obtained lncRNAs were mapped onto the *Sus scrofa* genome, using minimap2 [33], and only transcripts that had more than two exons were included in the analysis. Sample-distance correlation and the volcano plotting of DElncRNAs were performed, using the DESeq2 package. Differential clustering (heatmap) of DElncRNAs of each boar of either freezability group (GSF or PSF) was performed, using the Shiny-Seq R software package (https://schultzelab.shinyapps.io/Shiny-Seq) [34]. We further analyzed the lncRNAs splicing isoforms for length distributions, and screened the lncRNAs with the predictor analysis tools, CPC [30], coding potential assessment tool, CPAT (score < 0) and PFAM-Scan databases (OmicsBox software v.1.3.11) [35]. Potential splicing isoforms of lncRNAs obtained from three predictor analysis tools were used for Venn comparison analysis (Oliveros, 2007–2015; https://bioinfogp.cnb.csic.es/tools/venny) [36]. The Rfam biotype sequence distributions of the splicing isoforms of the lncRNAs were analyzed, using the OmicsBox software [35].

### 2.6. Functional Enrichment of Potential Target Genes of DElncRNAs

We assessed the functional role of the regulatory target genes of the DElncRNAs, using two independent algorithms, *cis* and *trans*. The *cis*-regulatory target genes were predicted within 10 kb window upstream and downstream of all the DElncRNAs, using the R Bioconductor packages [37]. The Pearson’s correlation coefficients were calculated between DElncRNAs and the corresponding target genes to predict their functional role in *trans*-regulation. Hierarchical clustering was used to visualize the expression profiles of the lncRNAs between the freezability groups and correlation analysis of the regulatory target genes of the lncRNAs, using the Circos software package [38]. We examined the correlation of a DElncRNA of each boar of the PSF group (TRINITY_DN1035446_c0_g1) or the GSF group (TRINITY_DN1094887_c21_g2) with the potential target gene. The functions of the lncRNA target genes were investigated in the Kyoto Encyclopedia of Genes and Genomes (KEGG) pathway [39]. The OmicsBox software was used to perform functional annotations of the potential protein-coding genes, according to the Gene Ontology (GO) categories including biological process, molecular function and cellular components [35]. The *Sus scrofa* (11.1) Ensembl database was downloaded from Ensembl BioMart Martview application to perform the GO enrichment analysis.

### 2.7. Quantitative RT-qPCR Analysis

The DElncRNA expression profiles of the transcriptome data (Supplementary Table S1) were confirmed using RT-qPCR analysis. Glyceraldehyde-3-phosphate dehydrogenase (GAPDH) was used as the reference gene [40]. Primers used for the DElncRNAs (TRINITY_DN1365027_c0_g1_i2, TRINITY_DN1103286_c5_g1_i5 and TRINITY_DN1278737_c0_g1_i1) are shown in Table 1. Total RNA samples, isolated from the spermatozoa of each boar [14], were reversely transcribed, and cDNA was synthesized in a PCR Thermal Cycler (Labcycler, Sensoquest GmbH, Göttingen, Germany). For quantitative real-time analysis, 100 ng RNA was used as the template, and reactions were performed using the High Fidelity cDNA Synthesis Kit (Roche Diagnostics International, Basel, Switzerland) with random hexamer, according to the manufacturer’s recommendations. Real-time measurements of the amplification products were performed in a Real-Time PCR system (ABI 7900 H T, Applied Biosystems, CA, USA) [41]. Briefly, the master mix volume comprised 5 µL SYBR Green mix (Maxima SYBR Green/ROX qPCR Master Mix x2, Thermofisher Scientific, USA), 10 µM each forward and reverse primers (2 µL) and 3 µL template cDNA (equivalent amount of 3.75 ng mRNA). The relative quantification of the transcript expression between the freezability groups was measured, using the Real-Time PCR Miner algorithm [42].

### 2.8. Statistical Analysis

Statistical analysis was analyzed with the Statistica software package, version 13.0 (TIBCO Software Inc., CA, USA; StatSoft Polska, Kraków, Poland). The ANOVA assumption (Shapiro–Wilk W-test) was used to check the normality of the data distribution and the Levene’s test was used to examine for homogeneity of variance. ANOVA analysis was performed with the general linear modeling (GLM) procedure. The following linear model was used to analyze the effect of the boar on the fresh semen quality or PT semen quality traits, as described in a recent study [43].

Yij = µ + βi + eij, where Y is the measured semen quality traits; µ is the overall mean of each trait; βi is the fixed effect of boar; and eij is the random residual effect.

Multiple comparisons were performed with the Neuman–Keuls post hoc test, while comparisons between the GSF and PSF groups were analyzed with an independent two-tailed T-test. Results of the semen quality parameters are expressed as the mean ± SEM. The Mann–Whitney U test was used to compare the relative abundance of DElncRNAs between the freezability groups. Significance differences were considered at *p* < 0.05.

## 3. Results

### 3.1. Semen Quality Assessment

**Figure 1:**
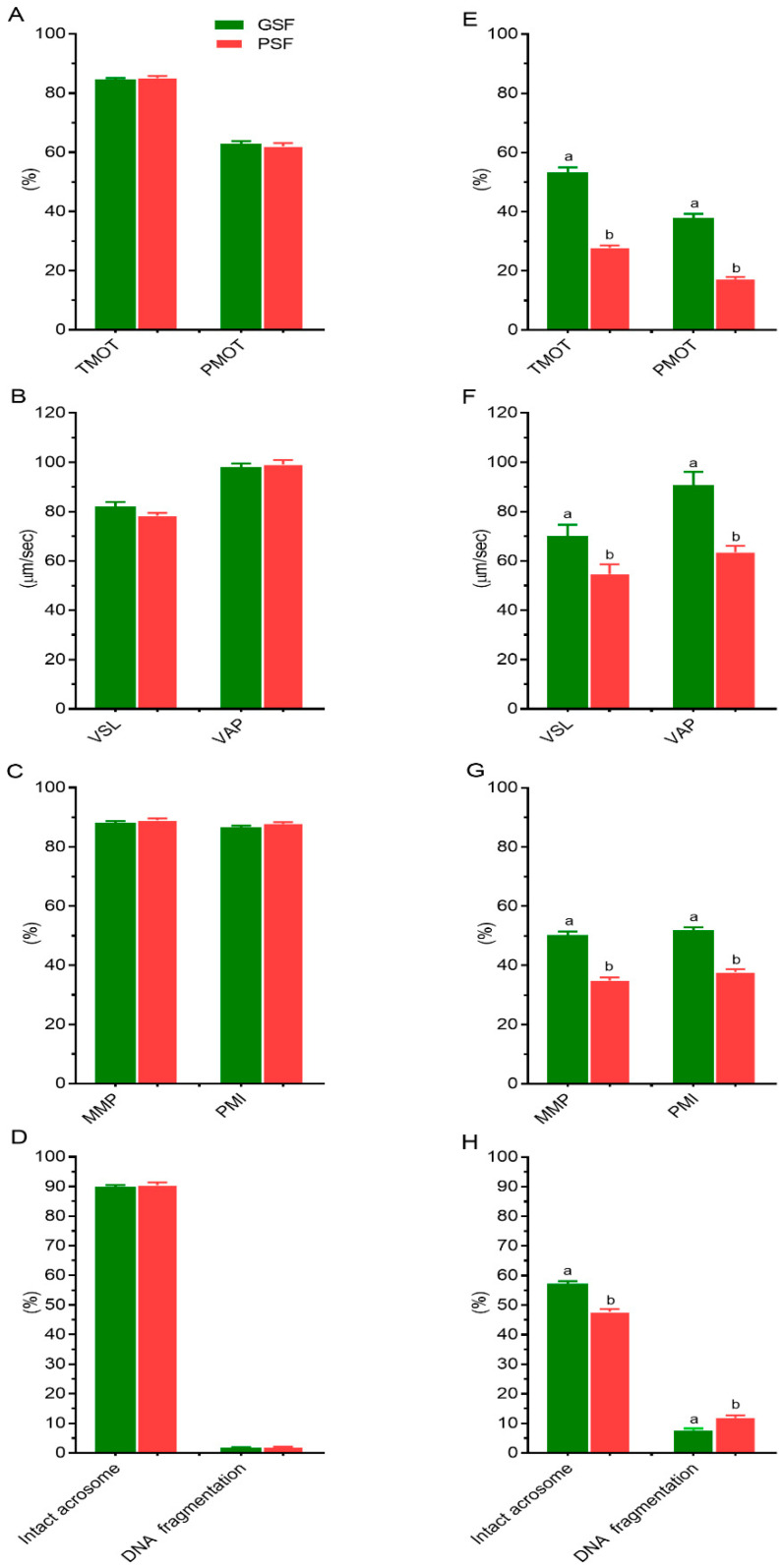
Motility (**A**,**E**), velocity (**B**,**F**) and (**C**,**D**,**G**,**H**) membrane integrity characteristics of boar spermatozoa with different freezability. Figures A–D represent fresh semen, while (**E**–**H**) represent post-thaw (PT) semen. Values are expressed as the mean (±SEM) of 26 and 33 ejaculates from boars classified as having good and poor semen freezability (GSF and PSF, respectively). Values with different letters (a,b) are significant at *p* < 0.05. TMOT—total motility; PMOT—progressive motility; VSL—velocity straight line; VAP—velocity average path; MMP—mitochondrial membrane potential; PMI—plasma membrane integrity.

Analysis of variance (ANOVA) showed that there were no significant (*p* > 0.05) effects on the fresh semen quality, represented by CASA-analyzed sperm motility and velocity parameters, and membrane integrity characteristics (Figure 1A–D). ANOVA results confirmed significant boar effects (*p* < 0.05) on post-thaw (PT) semen quality (Table 2). According to the PT analysis, four boars (G01, G04, G09 and G17) were classified as having good semen freezability, GSF (>30% TMOT) and the other five boars (P30, P36, P37, P38 and P39) were considered as having poor semen freezability, PSF (Figure 1). Besides PT sperm TMOT, boars of the GSF group exhibited higher (*p* < 0.05) PMOT (Figure 1E), VSL and VAP (Figure 1F) and membrane integrity, represented by MMP, PMI (Figure 1G), NAR acrosome integrity and DNA fragmentation (Figure 1H) than boars of the PSF group.

### 3.2. De Novo Transcriptome Assembly and Blast Statistics

**Figure 2:**
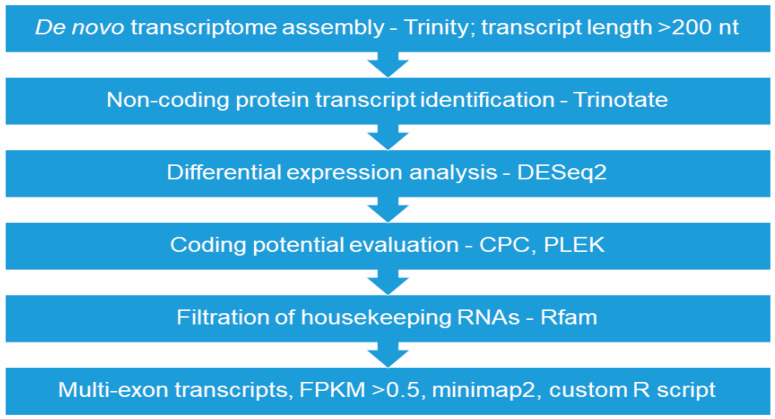
An overview of the pipeline used for the identification of long non-coding RNAs (lncRNAs) in boar spermatozoa. A total of 2,023,225 transcripts were obtained following de novo transcriptome assembly (Trinity); nt—nucleotides; Trinotate used as an annotation tool; DESeq2—differential expression tool; CPC (coding potential calculator) and PLEK (predictor of long non-coding RNAs and messenger RNAs based on an improved k-mer scheme) were used as the non-coding predictor tools; Rfam—non coding RNA database; FPKM—fragments per kilobase of transcripts per million reads mapped.

The summary statistics of *de novo* transcriptome assembly is shown in Table 3. For boar spermatozoa, the transcriptome assembly allowed the identification of a total of 2,023,225 trinity transcripts comprising about 36.49 GC content. It was found that the total assembled bases for all transcript contigs and for the longest isoform per gene were 715,830,046 and 667,299,497, respectively. An overview of the pipeline used to identify lncRNAs in boars of the freezability groups and remove protein-coding transcripts is shown in Figure 2. The pipeline predicted lncRNAs and validated them using the non-coding predictor analysis tools (CPC and PLEK), as indicated in Figure 2.

### 3.3. Gene Ontology (GO) Mapping, Annotations and Visualization of lncRNAs

**Figure 3:**
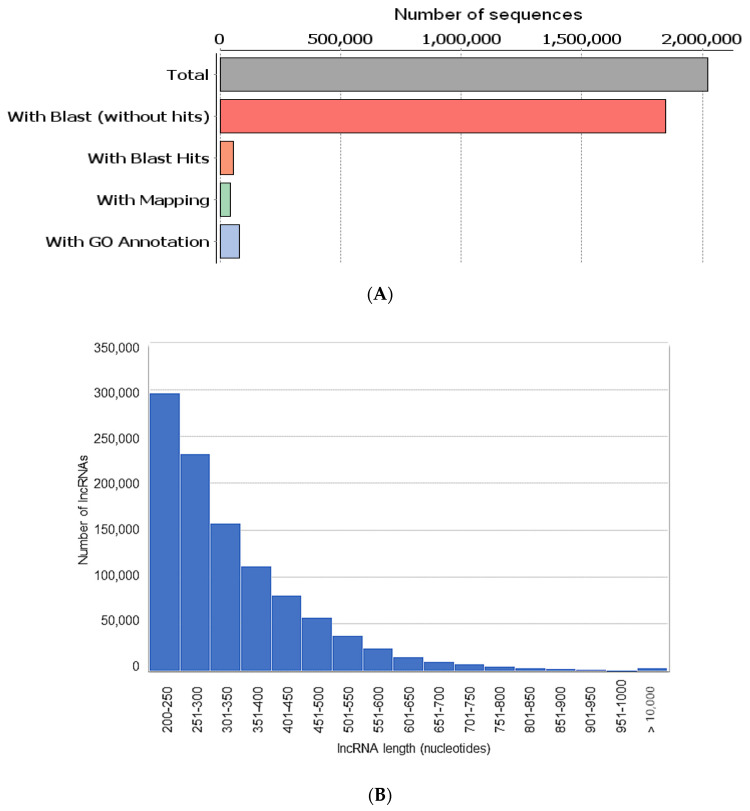
Sequence features of the long non-coding RNAs (lncRNAs) in boar spermatozoa. (**A**) Data distributions showing Blast hits, mapping and Gene Ontology (GO) annotation summary of the assembled contigs obtained from annotations with Blast2GO in the OmicsBox software. (**B**) Distributions of lncRNAs with length for Blast sequences.

Among the blasted sequences, 1,847,083 (91%) were without Blasts, 54,946 (3%) were with Blast hits, 41,777 (2%) were with GO mapping and 79,471 (4%) were with GO annotation (Figure 3A). A representative profile of the length distribution of the lncRNAs, with more than 200 nt in length (>200 nt), is shown in Figure 3B. We found that the length of the assembled transcriptome transcripts ranged from 200 to 2840 nt (Figure 3B).

**Figure 4:**
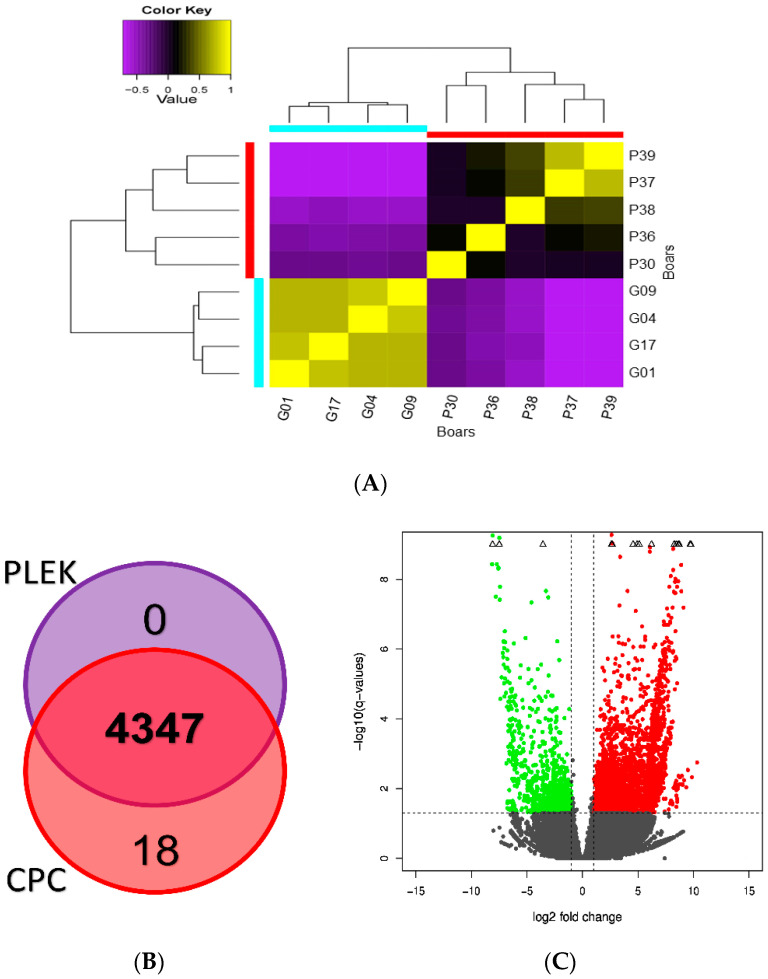
Sample-to-sample similarity analysis, Venn diagram and volcano plot of the long non-coding RNAs (lncRNAs) in boar spermatozoa: (**A**) sample-to-sample distance analysis of lncRNAs of four boars with good semen freezability (GSF), represented by G01, G04, G09, and G17, and five boars with poor semen freezability (PSF), indicated by P30, P36, P37, P38 and P39. Different colors indicate a normalized matrix with yellow representing high correlation; (**B**) Venn diagram of the identified potential lncRNAs in boar spermatozoa. Two predictor tools, CPC (score < −1) and PLEK (score < 0) were used to analyze the coding potential; (**C**) volcano plot showing expression levels of lncRNAs between the GSF and PSF groups. Each point represents an individual lncRNA. Black dots represent the lncRNAs that were not significantly differentially expressed. Red color indicates upregulation, while the green color indicates downregulation (log2fold change ≥ 1.0). The volcano plot represents the logarithmic (−log10) scale of the adjusted *p*-values shown in the *Y* axis and the logarithmic (log2) scale of the fold change shown in the *X* axis. An expression that is not within the scale plot is indicated in a diamond symbol.

A heat map of the sample-to-sample distance analysis showed the similarities and dissimilarities in lncRNA expression profiles among boars (Figure 4A). Boars of the GSF group were highly clustered in group, while at least three boars of the PSF group (P37, P38 and P39) were grouped together (Figure 4A). Boars of the GSF group exhibited higher similarity than the PSF group (Figure 4A). Comparison analysis with the Venn diagram showed the intersection results of 4347 lncRNAs from the non-coding predictor tools (Figure 4B). The sequences of the 4347 lncRNAs were further analyzed at the Rfam database where 4007 transcripts were predicted to be potential candidate lncRNAs for subsequent analysis (Figure 2).

**Figure 5:**
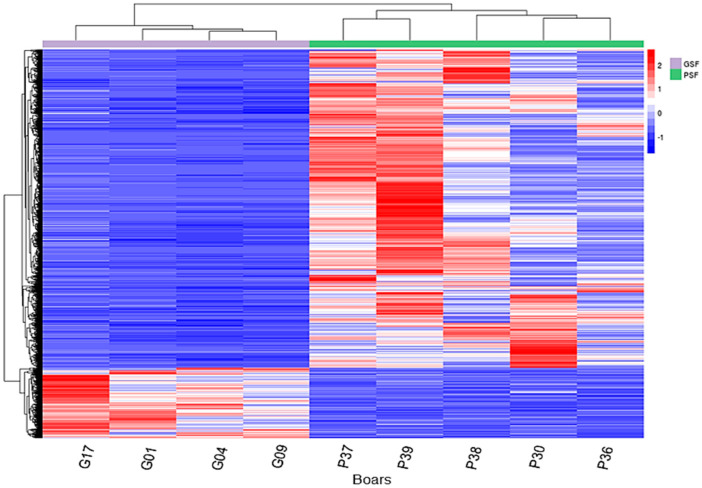
Heatmap clustering analysis of transferred expression values of DElncRNAs in individual boars with good and poor semen freezability (GSF and PSF, respectively). Boars of the GSF group are represented by G01, G04, G09, and G17, and boars of the PSF group are represented by P30, P36, P37, P38 and P39. The color scale in the hierarchical clustering map indicates the normalized counts output data. Red and blue colors indicate upregulation and downregulation expression profiles of DElncRNAs, respectively.

Visualization using the volcano plot (Figure 4C) showed significant variations in the expression levels of the lncRNAs between the freezability groups (PSF and GSF). We obtained a total of 4007 DElncRNAs, in which 3383 were upregulated in boars of the PSF group (Supplementary Table S1A) and 624 were downregulated in boars of the GSF group (Figure 4C, Supplementary Table S1B). In addition, the hierarchical clustering of the expression profiles confirmed the upregulation of a significant number of DElncRNAs in each boar of the PSF groups than in the GSF group (Figure 5).

### 3.4. Functional Analysis of Regulatory Target Genes of DElncRNAs

**Figure 6:**
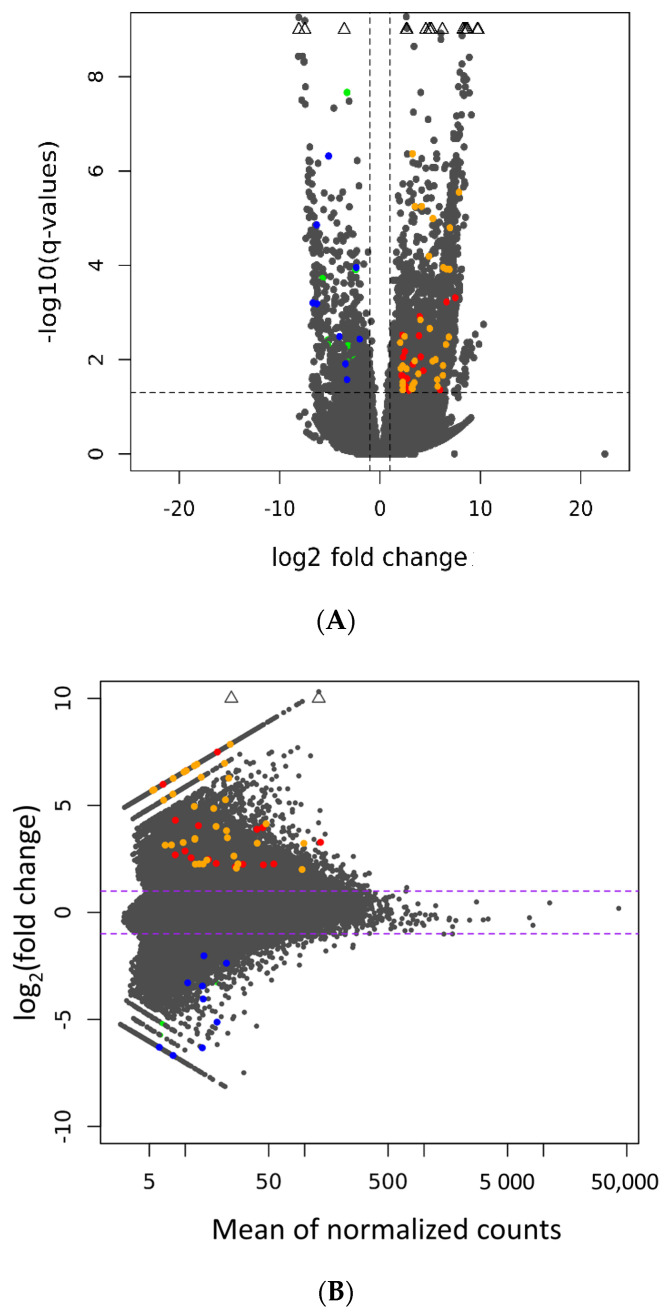
Visualization of differentially expressed long non-coding RNAs (DElncRNAs) and their potential regulatory target genes (**A**) Volcano plot. The volcano plot represents the logarithmic (−log10) scale of the adjusted *p*-values shown in the *Y* axis and the logarithmic (log2) scale of the fold change shown in the *X* axis. (**B**) MA plot. The MA plot represents the logarithmic scale of the fold change shown in the *Y* axis and the normalized expression count values shown in the *X* axis. Orange and blue dots represent upregulated and downregulated DElncRNA, respectively. Red and green dots represent upregulated and downregulated target genes, respectively. The expression that is not within the scale plot is indicated in a diamonds symbol.

We predicted that the upregulation of 35 DElncRNAs in boars of the PSF group and the downregulation of nine DElncRNAs in boars of the GSF group were associated with *cis*- and *trans*-regulatory target genes (Supplementary Table S2). In addition, we predicted 25 potential *cis*-regulated target genes, in which 17 were detected in the PSF group (Table 4), and eight were identified in the GSF group (Table 5). Furthermore, it was found that some DElncRNAs exhibited dual-regulatory roles of protein-coding genes in *cis* and *trans* (Table 4 and Table 5). The expression data of the DElncRNAs and their potential target genes are illustrated in the volcano (Figure 6A) and MA plots (Figure 6B).

**Figure 7:**
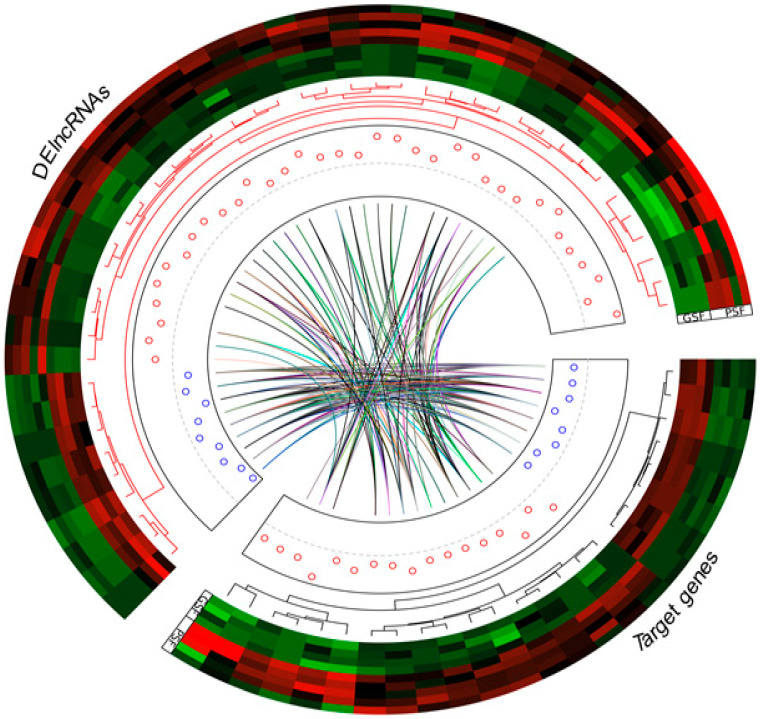
Circos plot showing expression patterns in relation to the differentially expressed long non-coding RNAs (DElncRNAs with their potential target genes, and co-expression interactions. Circular tracks from outside to inside: nine outermost tracks indicate hierarchical clustering with dendrograms of DElncRNAs and regulatory target genes (normalized Z-score expression values) of boars classified as having good and poor semen freezability (GSF and PSF, respectively). Red and green colors indicate upregulation and downregulation, respectively. Inner tracks with scatter plots (circles) represent log2 fold change values (red and blue circles indicate upregulation and downregulation, respectively). Middle track represents the co-expression of the DElncRNAs with *cis*- and *trans*-regulatory targets.

**Figure 8:**
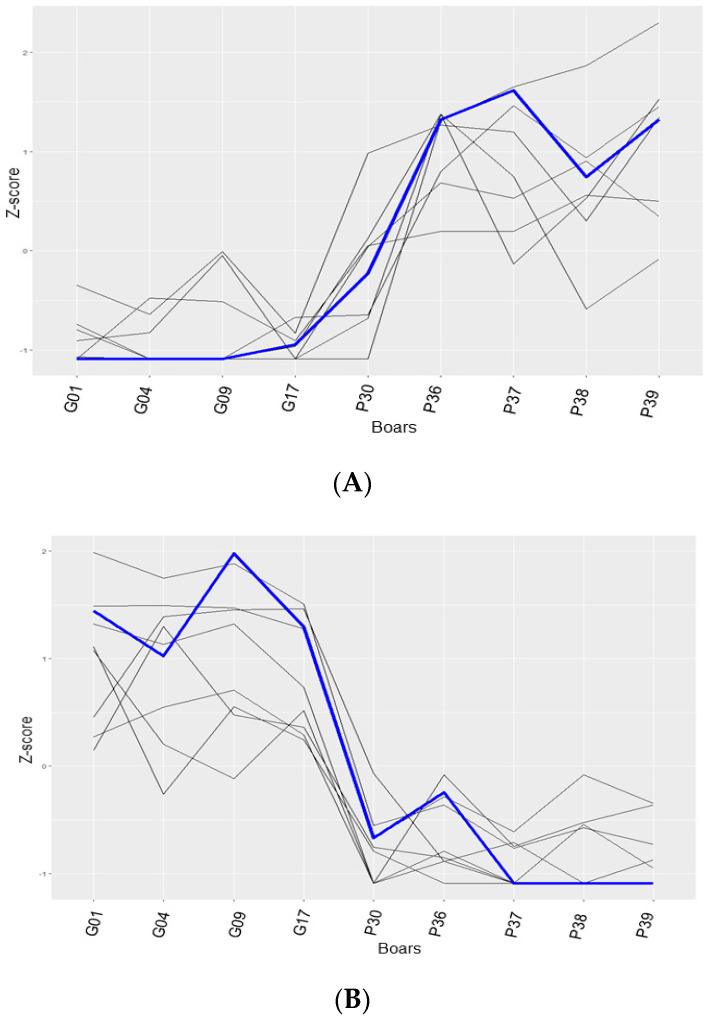
Correlation analysis of the differentially expressed lncRNAs (DElncRNAs) with potential target genes. (**A**) Positive correlation of an upregulated DElncRNA (TRINITY_DN1035446_c0_g1) of the poor semen freezability (PSF) group with its target gene. (**B**) Positive correlation of a downregulated DElncRNA (TRINITY_DN1094887_c21_g2) of the good semen freezability (GSF) group with its target gene. The Z-score represents the normalized values of the expression data. The blue line represents the target gene, while the black lines indicate the DElncRNAs. Four boars with GSF are represented by G01, G04, G09, and G17, while five boars with PSF are represented by P30, P36, P37, P38 and P39.

The Circos plot shows the highly variable DElncRNAs between the freezability groups (PSF and GSF), the expression profile data of the regulatory target genes and their corresponding DElncRNAs, and co-expression interactions (Figure 7). The Circos plot displays differences in the expression patterns of the DElncRNAs in relation to their corresponding regulatory target genes. Significant interactions (Pearson’s correlation, r ≥ 0.70, *p* < 0.05) were found between the DElncRNAs and the regulatory target genes (middle track in Circos plot). We provided two examples of the expression correlation analysis of the DElncRNAs with their potential target genes of the PSF (Figure 8A) and GSF groups (Figure 8B).

Predictive analysis showed that the DElncRNAs of the PSF group had potential *cis*- or *trans*-regulatory effects on several different protein-coding genes, such as *COX7A2L*, *TXNDC8* and *GAS2* (Table 4). Likewise, DElncRNAs from boars of the GSF group had potential *cis*- or *trans*-regulatory effects on different protein-coding genes, such as *ROBO2* and *SOX-7* (Table 5). The protein target genes that are associated with different reproductive traits or sperm functions are presented in Table 6. In the literature, we did not find any potential role of *CNTNAP5,* contactin associated protein like 5 (ENSSSCG00000015726) and *CAAP1,* caspase activity and apoptosis inhibitor 1 (ENSSSCG00000005127) in sperm functions.

### 3.5. Splicing Isoforms of lncRNAs

We detected approximately 124,543 sequences with potential lncRNA splicing isoforms, in which 13,234 showed splicing events within a single locus. Comparison analysis with the Venn diagram showed that the intersection results from the three predictor analysis tools (CPAT, CPC and PFAM-Scan databases) yielded about 12,200 lncRNAs with potential splicing isoforms (Supplementary Figure S1A). The average sequence distribution of the spliced isoforms was 385 nt (Supplementary Figure S1B). Furthermore, we detected approximately 69 lncRNA splicing isoforms with Rfam biotype sequences(Supplementary Figure S1C).

### 3.6. Functional Annotations of Potential Target Genes of DElncRNAs

**Figure 9:**
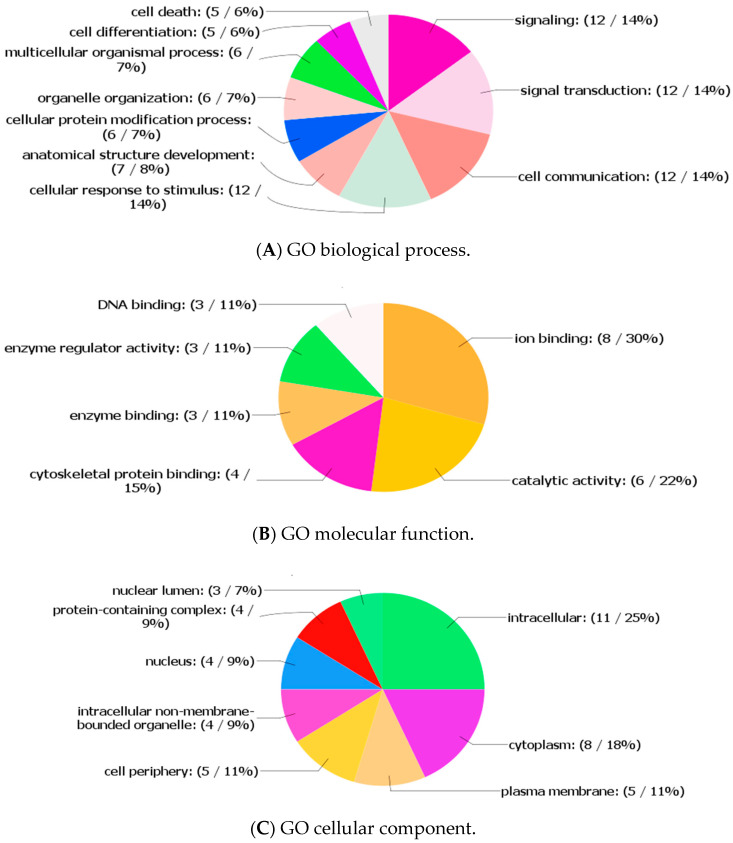
Gene Ontology (GO) enrichment analysis of potential protein-coding genes of differentially expressed long non-coding RNAs (DElncRNAs): (**A**) GO biological process; (**B**) GO molecular function; and (**C**) GO cellular component. The value in the parenthesis displays the number of inputs and the percentage of target genes enriched in the category.

To predict the regulatory functions of the DElncRNAs on sperm freezability we performed the Kyoto Encyclopedia of Genes and Genomes (KEGG) pathway and Gene Ontology (GO) analyses for the target protein-coding genes that were predicted to co-express with the DElncRNAs in *cis* and *trans*. According to the KEGG pathway analysis, the target genes were assigned to different pathways such as JAK–STAT (ssc04630) for *GHR* gene (*p* < 0.08) and axon guidance pathway (ssc04360) for *ROBO2* gene (*p* < 0.09). Enrichment analysis demonstrated that the GO terms of the target genes were associated with various molecular function categories, biological process categories and cellular component categories (Supplementary Table S3). The GO terms related to the enriched target genes are shown in biological process categories (Figure 9A), mitochondrial function categories (Figure 9B), cellular component categories (Figure 9C). We did not detect any GO terms for *CAAP1* and *NUPR2*. It was found that ENSSSCG0000033794 is a lncRNA and ENSSSCG00000040848 is a U6 spliceosomal RNA, which are non-protein coding genes that appeared to have a *cis*-regulatory role. We did not detect ENSSSCG0000033600, ENSSSCG00000038136 (novel), ENSSSCG00000034138 (novel) and ENSSSCG00000023812 in the *Sus scrofa* 11.1 reference genome.

### 3.7. Quantitative Reverse Transcription Polymerase Chain Reaction (RT-qPCR) Analysis

**Figure 10:**
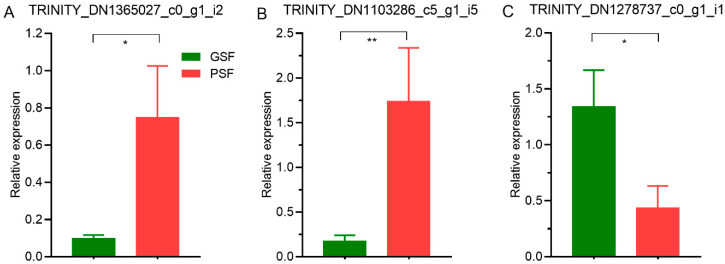
The validation of expression patterns of long non-coding RNAs in boars with poor and good semen freezability (PSF and GSF, respectively) using RT-qPCR analysis: (**A**,**B**) upregulated DElncRNA transcripts (TRINITY_DN1365027_c0_g1_i2 and TRINITY_DN1103286_c5_g1_i5, respectively) in the PSF group, and (**C**) an upregulated DElncRNA transcript (TRINITY_DN1278737_c0_g1_i1) in the GSF group. LncRNA expression was quantified relatively to the expression of glyceraldehyde-3-phosphate dehydrogenase (GAPDH). Significant differences, * *p* < 0.05, ** *p* < 0.01.

The RT-qPCR analysis showed significant (*p* < 0.05) differences in the expression levels of the DElncRNAs between the PSF and GSF groups (Figure 10A–C), similarly to the *de novo* assembled transcriptome expression profiles (Supplementary Table S1). It was confirmed that the expression of upregulated DElncRNAs (TRINITY_DN1365027_c0_g1_i2 and TRINITY_DN1103286_c5_g1_i5), obtained by RT-qPCR, was significantly higher (*p* < 0.05) in the PSF group (Figure 10A,B). Likewise, the expression of a downregulated DElncRNA (TRINITY_DN1278737_c0_g1_i1) was significantly higher (*p* < 0.05) in the GSF group than in the PSF group (Figure 10C).

## 4. Discussion

### 4.1. Semen Freezability

The freezability of boar semen is dependent on many factors, including individual variability [14,62,63]. Studies in our laboratory [14,17,19,22] and those of other findings [62,63] have shown that boar spermatozoa differ significantly in their response to cryopreservation conditions; they are classified as having good freezability and poor freezability ejaculates based on the PT semen quality. A battery of sperm traits that provides reliable estimates of the sperm viability has been routinely used to assess boar semen freezability [14,17,19,63,64,65].

### 4.2. Functional Characteristics of Potential Target Genes of DElncRNAs

In this study, we used nine biological replicates, four and five replicates for the GSF and PSF groups, respectively. Furthermore, significant differences in the *de novo* assembled transcriptome expression levels of the DElncRNAs between the freezability groups were confirmed by RT-qPCR analysis. To assess the effect of DElncRNAs on sperm freezability, we profiled the DElncRNA expression profiles into two freezability groups and detected an abundance of upregulated DElncRNAs in spermatozoa from the poor freezability ejaculates. Previous studies reported that the presence of abundant lncRNA transcripts in mature spermatozoa might have potential functions in these cells [9,11,66]. Moreover, evidence has indicated that the presence of abundant coding and non-coding RNA transcripts in stallion spermatozoa is not a random remnant from spermatogenesis but represents a selectively retained and functionally coherent collection of RNAs [66]. Accordingly, specific subsets of lncRNA transcripts have been suggested to play regulatory roles in spermatogenesis [1,9,10] and the dysfunction of the lncRNA-related mechanisms involved in spermatogenesis might result in reduced sperm concentration and fertility [2]. Recently, it has been demonstrated that lncRNAs located on cattle quantitative trait loci (QTLs) were associated with sperm motility, and that the expression profiles of lncRNAs differed between spermatozoa with high and low motility [67]. Presumably, the presence of abundant lncRNAs in boar spermatozoa indicates that the DElncRNAs are implicated in important regulatory roles in the functions of spermatozoa, thereby influencing their response to the cryopreservation conditions.

Accumulating evidence has shown that lncRNAs could regulate protein-coding genes that are implicated in spermatogenesis, fertility and embryo development [1,3,4,10,67]. The transcriptional regulation of lncRNAs could be predicted as *cis* and *trans*, and could have a positive or negative effect on gene expression [7]. It is noteworthy that lncRNA regulates the expression of a target gene by *cis*-regulation, which is restricted to the chromosome from which it is transcribed, while a lncRNA role in *trans*-regulation is predicted when it affects genes on other chromosomes at the expression level [6,7]. We hypothesized that the DElncRNA target genes could act through *cis*- or *trans*-regulating mechanisms, thereby affecting sperm freezability. Our findings reaffirm those of a previous study, indicating that lncRNAs are involved in the regulation of sperm cryo-tolerance [11]. Interestingly, we found that a few DElncRNAs were *cis*-regulators and *trans*-regulators in boar spermatozoa. Such findings confirm those of previous reports, indicating that some *cis*-regulating lncRNAs could act in trans, reinforcing the concept that lncRNAs do not have strict sequence conservation restraints [1,68,69].

Enrichment analysis confirmed that twenty-four potential target genes were related to a wide variety of GO terms. Signaling (GO:0023052), signal transduction (GO:0007165), cell communication (GO:0007154) and cellular response to stimulus (GO:0051716) were the top four listed GO terms of the biological process categories. Ion binding (GO:0043167) and catalytic activity (GO:0003824) were the top two listed GO terms of the mitochondrial function categories and intracellular (GO:0005737) was predominant in the cellular component categories. It was found that the GO terms “signaling” and “signal transduction” were related to several protein-coding genes, such as *GHR*, *THRB* and *RGS18,* which are predicted the target genes of upregulated DElncRNAs in *cis*. Although we did not detect a significantly enriched KEGG pathway, the association of *GHR* with the signaling cascades, such as JAK–STAT, reaffirms the gene role in sperm functions. According to the study of Ran et al. [11], sperm transcripts related to apoptotic-related signaling pathways, such as JAK–STAT and the P13K–Akt cascade, might have a regulatory role in apoptosis during cryopreservation. The *GHR* is a member of the cytokine receptor superfamily, and *GH* has multiple specific effects on the male reproductive physiology, including steroidogenesis and spermatogenesis [44]. Notably, the testis-specific cells are potential targets for direct and indirect actions of *THRB*, a receptor for the thyroid hormone (TH) which regulates the proliferation and differentiation of the Sertoli cells and Leydig cells during testicular development [45,70]. Moreover, a previous study showed that the thyroid hormone, represented by 3,5,3′-triiodothyronine (T_3)_ and thyroxine (T_4_), affected DNA integrity by increasing reactive oxygen species (ROS) production [71]. However, in another study, it was reported that membrane-bound receptors for hormones, cytokines, growth factors or neurotransmitters are implicated in various sperm functions, such as motility and capacitation [72]. Moreover, *RGS18* belongs to the G-protein signaling family, which inhibits signal transduction by increasing the GTPase activity of G protein alpha subunits [73] and it is expressed in the testis [74]. Our findings suggest that the co-expression of *cis*-regulating DElncRNA with *GHR*, *THRB* or *RGS18* was associated with increased sperm cryo-damage, however, the functions of these genes in sperm freezability are unclear.

Interestingly, some predicted target genes (*GHR, THRB, CDC73*, *RAB31*, *ULK4* and *EEPD1*) of the PSF group were related to GO terms such as “cellular response to stimulus” and “response to stress”, reaffirming the regulatory role of DElncRNAs in sperm functions. In addition to the regulatory role of lncRNAs, it has been reported that circular RNAs (circRNAs) could also influence the function of *CDC73*, which is implicated in embryo development [46]. It should be noted that *CDC73* is a target gene of upregulated DElncRNA in *cis*, while *SOX-7*, which is implicated in embryo development [47], is a *trans*-regulatory gene. However, it is unclear how the regulatory effects of these genes could affect sperm cryo-tolerance in either freezability group. It is noteworthy that *RAB31* belongs to the RAS protein family that plays important roles in various cellular functions, including protein trafficking, transmembrane signal transduction and autophagy [48]. Notably, the RAB-related autophagy gene could promote apoptosis and its overexpression was associated with poor sperm cryo-survival [14]. In addition to its association with response to stress, *ULK4* is implicated in various biological processes, such as motility and cytoskeleton assembly and probably acts as an essential scaffold protein regulating ciliogenesis [46,75]. Likewise, *COX7A2L*, a predicted target gene of the upregulated DElncRNA in *trans*, is implicated in sperm motility [49]. We suggest that the functional relevance of the co-expression of *ULK4* and *COX7A2L* in sperm freezability is not clearly understood. However, previous studies reported that cytoskeletal microtubules and dynein motor proteins could be implicated in autophagy-related processes, which cooperate with apoptosis to maintain cellular survival functions in response to stressful conditions [76,77]. More importantly, *EEPD1,* another protein-coding gene of the upregulated DElncRNA in *cis,* is a replication stress-response gene that is implicated in the maintenance of genome stability [50]. We hypothesize that the overexpression of the stimulus- and stress-related target genes of DElncRNAs of the PSF group could exacerbate the sperm’s response to the cryopreservation conditions, resulting in reduced cryo-survival. Such findings seem to be consistent with the function of *TXNDC8,* a *cis*-regulatory DElncRNA of the PSF group. Notably, *TXNDC8* is highly expressed in immature spermatozoa, which protects sperm cells against oxidative stress and its expression in mature spermatozoa is suggested as a potential marker for male infertility [51]. In a previous study, it has been suggested that increased levels of antioxidant enzymes in frozen–thawed (FT) boar semen might be due to the protective response of the sperm cells to cold stimulation and oxidative stress [78]. More recently, it has been reported that *cis*- or *trans*-regulating lncRNAs are implicated in the regulation of the immune response, and the antioxidant system is activated in response to increased oxidative stress [79]. It is likely that the co-expression of upregulated DElncRNA with *TXNDC8* in mature spermatozoa could be in response to increased oxidative stress during spermatogenesis in boars of the PSF group. Even though the molecular mechanisms currently remain in terms of the action of the stimulus- and stress-related target genes on sperm functions, we speculate that the co-expression of these genes with the DElncRNAs could predispose spermatozoa of the PSF group to increased cryo-damage.

Of interest is the co-expression of DElncRNA and potential target genes, such as *MAST2*, *TTLL7, TECTA*, *EFCAB11* (*EFCAB2*) and *GAS2*, which are associated with cytoskeletal regulation and assembly. It is noteworthy that *MAST2*, a microtubule-associated serine/threonine kinase, is associated with microtubules in the spermatid manchette, which takes place in spermatid maturation during spermiogenesis [52]. Studies have demonstrated the significant role of the *TTLL*-mediated pathway for α-tubulin polyglutamylation [53], and the *TECTA* protein in the assembly and regulation of axoneme [54]. However, the functional implications of *TTLL7* and *TECTA* proteins in sperm functions during cryopreservation are currently unclear. Moreover, the expression of *EFCAB11* was detected in spermatogenic cells, and the identification of the *EFCAB11* protein in the principal piece of the flagellum of mouse spermatozoa indicates its role in the regulation of flagellar movement [55]. Another target gene of interest, *GAS2*, appears to play a role in microfilament organization, and might modulate the cell susceptibility to apoptosis [56]. However, whether the *GAS2* protein is an indispensable component of the apoptotic machinery in spermatozoa during cryopreservation remains to be determined because cryo-induced apoptotic-like changes in FT spermatozoa have been shown to compromise their functions [14,15,63]. It is noteworthy that the cytoskeletal-associated genes are implicated in the functions of the flagellar movement of spermatozoa, however, the overexpression of the cytoskeletal proteins could compromise tubulin stability [56]. In the present study, the cytoskeletal-associated genes of the DElncRNAs in *cis* or *trans* were mostly observed in the poor freezability ejaculates, suggesting that the co-expression of these genes with the DElncRNAs could compromise the functions of spermatozoa, rendering them more susceptible to cryo-induced injury.

Remarkably, a significantly enriched KEGG pathway was not detected for *ROBO2*, which is associated with the axon guidance pathway [80]. We have shown in our recent study that the *ROBO1* is significantly associated with the axon guidance pathway [43], which appears to have a potential biological function in boar fertility [57]. It is noteworthy that *ROBO2* receptors have been detected in the testis, and their interaction with a secretory protein (SLIT) contributes to the SLIT–ROBO signaling pathway that is implicated in cell adhesion and cell death [80]. However, *ROBO2* expression was exerted by the *cis*-regulating DElncRNA of the GSF group and was associated with the co-expression of *trans*-regulating effects of DElncRNA on *NUPR2*. Although there is no literature regarding the role of *NUPR2* in sperm function, evidence has shown that *NUPR1* is a multifunctional protein that interacts with several signaling pathways, such as the PI3K/AKT signaling pathway [81]. Mounting evidence suggests that lncRNAs are involved in sperm function by acting on protein-coding genes through *cis*-acting elements and *trans*-acting factors [9,10,11]. Our observations indicate that the co-expression of downregulated DElncRNAs with *ROBO2* and *NUPR2* was associated with the reduced cryo-damage to spermatozoa, thereby reaffirming the important role of lncRNAs in *cis*- and *trans*-regulations of sperm functions.

More importantly, *CD163L1* and *ITPRID1* are *cis*-regulated genes of DElncRNAs of the PSF group, whereas protocadherin alpha-C2 and *ZMAT4* are *cis*-regulated genes of DElncRNAs of the GSF group that are integral membrane components which are implicated in different biological processes, such as cell–cell recognition, cell adhesion, apoptosis and metabolism [58,59,60,82,83]. Evidence has shown that *CD163L1* functions as a scavenger receptor for one or several ligands associated with inflammation [58] and is required to maintain the immunoprivileged environment in the testis [82]. Even though *ITPRID1* belongs to the sperm-specific antigen 2-related protein family that is involved in fertilization [83], there is no specific role of this gene in sperm functions in the current literature. It should be emphasized that protocadherins are implicated in spermatogenesis and fertilization [59], while *ZMAT4* is associated with bull fertility and is involved in apoptotic, biological, developmental and metabolic processes [60]. The findings of these previous studies show that the cell–cell recognition genes perform a diverse set of functions in the male reproductive tract [58,59,60,82,83]. Furthermore, our results suggest that the co-expression of upregulated DElncRNA with *CD163L1* or *ITPRID1* was concurrent with poor sperm cryo-survival. In contrast, the co-expression of downregulated DElncRNA with either protocadherin alpha-C2 or *ZMAT4* was associated with higher sperm cryo-tolerance. Presently, we are unable to explain these findings and further studies are warranted. Along with its role in prostate cancer, the high expression of *KLK15* confirms that the gene is implicated in spermatogenesis [61]. However, the mechanism by which the co-expression of upregulated DElncRNA with *KLK15* affected sperm freezability remains unclear and would require further investigations.

We detected splicing isoforms of lncRNAs in boar spermatozoa and provided limited results on their characteristic features (Supplementary Figure S1). Several modes of alternative transcript events have been identified in human and mouse genes [84]. There is evidence indicating that a higher proportion of alternative splicing events in spermatozoa was detected in lncRNA than in the mRNA [9], however, lncRNA splicing events were not fully investigated in this study. Even though it has been suggested that lncRNAs can regulate the alternative splicing of pre-mRNA by various mechanisms, including interactions with the specific splicing factors [4,85], little is known about the regulatory roles of lncRNA spliced isoforms in spermatogenesis. Follow-up studies will be performed in our laboratory to determine more about the role of the splicing isoforms of lncRNAs in sperm functions.

## 5. Conclusions

Using *de novo* transcriptome assembly, we identified a catalogue of DElncRNAs, whose functions in boar spermatozoa remain largely unknown. Our findings show that a majority of the DElncRNAs was upregulated in the poor freezability ejaculates and appeared to significantly affect the sperm’s response to the cryopreservation conditions. Furthermore, we predicted that the potential target genes were *cis*- or *trans*-regulated by the DElncRNAs. The GO enrichment analysis confirmed that the target genes are associated with numerous biological processes, including signal transduction, response to stress, cell death (apoptosis), motility, reproduction and embryo development. Understanding the mechanism by which lncRNAs interact with their neighboring genes is essential to elucidate the role of sperm functions in cryo-tolerance. Further research studies are needed to provide more valuable insights into the mechanisms regarding the regulatory role of lncRNAs in sperm development processes to obtain a better understanding of their roles in semen freezability.

## Figures and Tables

**Table 1:** Primers used for the validation of differentially expressed long non-coding RNAs (DElncRNA) using quantitative reverse transcription polymerase chain reaction (RT-qPCR).

DElncRNA ID	Primer Sequence (5′-3′)	Temperature (°C)	Product Size (bp)
TRINITY_DN1365027_c0_g1_i2	F: ccatatgctgtgggtgagg R: ttgtgatggaggataatttgagaa	60	167
TRINITY_DN1103286_c5_g1_i5	F: aaaacagaaaggaaatgaaacca R: ggaaattaagccctcattgg	60	100
TRINITY_DN1278737_c0_g1_i1	F: catatttctttacatcatggtcctg R: tttagagcttcagtgatgtgc	60	150

**Table 2:** ANOVA showing boar effect on post-thaw (PT) semen quality.

Sperm Parameters (df = 8)	*F*-Value	*p*-Value
Total motility (TMOT)	34.641	<0.001
Progressive motility (PMOT)	27.753	<0.001
Velocity straight line (VSL)	16.916	<0.001
Velocity average path (VAP)	6.944	<0.001
Mitochondrial membrane potential (MMP)	8.785	<0.001
Plasma membrane integrity (PMI)	13.316	<0.001
Normal apical ridge (NAR) acrosome integrity	7.938	<0.001
DNA fragmentation	3.712	<0.006

Significantly at *p* < 0.05; df—degree of freedom.

**Table 3:** Summary statistics of de novo transcriptome assembly of 9 polish large white (PLW) boars.

Gene and Transcript Counts, and guanine-cytosine (GC) content	Total trinity ‘genes’:	1,879,557
	Total trinity transcripts:	2,023,225
	Percent GC	36.49
Statistics Based on All Transcript Contigs:	Contig N10 (nt):	660
	Contig N20 (nt):	534
	Contig N30 (nt):	464
	Contig N40 (nt):	411
	Contig N50 (nt):	366
	Median contig length (nt):	310
	Average contig (nt):	353.78
	Total assembled bases:	715,783,046
Statistics Based on Only The Longest Isoform per ‘gene’:	Contig N40 (nt):	662
	Contig N20 (nt):	535
	Contig N30 (nt):	466
	Contig N40 (nt):	413
	Contig N50 (nt):	367
	Median contig length (nt):	312
	Average contig (nt):	355.03
	Total assembled bases:	667,299,497

nt—nucleotides.

**Table 4:** Potential regulatory target genes of differentially expressed long non-coding RNAs (DElncRNAs) in the poor semen freezability (PSF) group. *n/a*—not available.

lncRNA ID	Locus	*Cis*-Regulation	*Trans*-Regulation
TRINITY_DN1035446_c0_g1	chr13:15027825-15028258	ENSSSCG0000033794	*CAAP1, GAS2, COX7A2L*
TRINITY_DN1044754_c0_g1	chr6:165435006-165435571	*MAST2*	*n/a*
TRINITY_DN1045510_c0_g1	chr16:27228522-27229268	*GHR*	*COX7A2L*
TRINITY_DN1361685_c0_g1	chr1:258475511-258475820	*U6*	*n/a*
TRINITY_DN1059433_c0_g1	chr11:14840949-14841766	*n/a*	*CAAP1*
TRINITY_DN157051_c1_g1	chr13:11234377-11234878	*THRB*	*COX7A2L, EFCAB11 (EFCAB2)*
TRINITY_DN1778675_c1_g1	chr6:99002552-99002962	*n/a*	*COX7A2L, KLK15, EFCAB11 (EFCAB2)*
TRINITY_DN1778721_c0_g1	chr1:146979892-146981163	*n/a*	*CAAP1*
TRINITY_DN241239_c0_g1	chr10:597871-598247	*CDC73*	*EFCAB11 (EFCAB2)*
TRINITY_DN263649_c0_g1	chr10:1545624-1546297	*RGS18*	*GAS2, COX7A2L*
TRINITY_DN4560_c0_g1	chr14:106830465-106891960	ENSSSCG0000033600	*n/a*
TRINITY_DN416076_c0_g1	chr7:94728590-94729707	*n/a*	*CAAP1*, *COX7A2L*
TRINITY_DN538320_c0_g1	chr5:63499679-63500175	*CD163L1*	*n/a*
TRINITY_DN559365_c0_g1	chr1:251338735-251339236	*TXNDC8*	*GAS2*
TRINITY_DN600110_c0_g2	chr18:37853087-37853635	*EEPD1*	*n/a*
TRINITY_DN625807_c0_g1	chr7:10417725-10418388	*n/a*	*CAAP1*, *EFCAB11 (EFCAB2)*
TRINITY_DN640486_c0_g1	chr18:41489497-41491323	*ITPRID1*	*CAAP1, GAS2*
TRINITY_DN740880_c0_g1	chr8:16645743-16646197	*n/a*	*CAAP1*, *COX7A2L*
TRINITY_DN848488_c0_g1	chr17:4530969-4531377	*n/a*	*COX7A2L*
TRINITY_DN869342_c0_g1	chr1:30107201-30107838	*n/a*	*EFCAB11 (EFCAB2)*
TRINITY_DN934504_c0_g1	chr6:98298308-98298817	*RAB31*	*n/a*
TRINITY_DN942459_c0_g1	chr13:109345030-109346972	n/a	*CAAP1*, *GAS2*
TRINITY_DN962891_c0_g1	chr6:129774162-129774633	*TTLL7*	*COX7A2L*
TRINITY_DN971351_c0_g1	chrX:6727378-6736952	*n/a*	*CAAP1*
TRINITY_DN976443_c0_g1	chr13:43295569-43296123	*n/a*	*COX7A2L, KLK15, EFCAB11 (EFCAB2)*
TRINITY_DN987550_c1_g1	chr13:25727243-25727879	*ULK4*	*COX7A2L*
TRINITY_DN1058184_c4_g1	AEMK02000598.1:14135-15082	ENSSSCG00000038136	*CAAP1*, *COX7A2L, KLK15*
TRINITY_DN792292_c0_g1	AEMK02000514.1: 18098-64313	ENSSSCG00000034138	*n/a*

**Table 5:** Potential regulatory target genes of differentially expressed long non-coding RNAs (DElncRNAs) in the good semen freezability (GSF) group.

lncRNA ID	Locus	*Cis*-Regulation	*Trans*-Regulation
TRINITY_DN1022003_c0_g2	chr2:142618997-142620529	*LOC100621701*	*TECTA*, *SOX-7, NUPR2*
TRINITY_DN1094887_c21_g2	chr13:177656907-177657455	*ROBO2*	*TECTA*, *SOX-7, NUPR2*
TRINITY_DN1225954_c0_g1	chr11:31386574-31386891	*n/a*	*TECTA*, *SOX-7, NUPR2*
TRINITY_DN1278737_c0_g1	chr6:2487124-24872979	*n/a*	*SOX-7, NUPR2*
TRINITY_DN652283_c0_g1	chr17:9809518-9810042	*ZMAT4*	*TECTA*, *NUPR2*
TRINITY_DN698757_c0_g1	chr6:13294125-132941663	*n/a*	*TECTA*, *NUPR2*
TRINITY_DN742894_c0_g1	chr3:60110813-60111197	ENSSSCG00000023812	*TECTA*, *SOX-7, NUPR2*
TRINITY_DN980890_c0_g1	chr15:27098747-27099086	*CNTNAP5*	*TECTA*, *NUPR2*

*n/a*—not available.

**Table 6:** Protein-coding genes of differentially expressed long non-coding RNAs (DElncRNAs) in boar spermatozoa. *n/a*—not available, no reference was found.

Ensembl	Gene Name	Gene Description	Sperm/Reproductive Traits	References
ENSSSCG00000016866	*GHR*	growth hormone receptor	steroidogenesis and spermatogenesis	[44]
ENSSSCG00000036033	*THRB*	thyroid hormone receptor beta	steroidogenesis	[45]
ENSSSCG00000033945	*RGS18*	regulator of G protein signaling 18	uncharacterized	*n/a*
ENSSSCG00000010801	*CDC73*	cell division cycle 73	embryo development	[46]
ENSSSCG00000025408	*ULK4*	unc-51 like kinase 4	motility	[46]
ENSSSCG00000031889	*SOX-7*	SRY-box transcription factor 7	embryo development and implantation	[47]
ENSSSCG00000037264	*RAB31*	RAB31, member RAS oncogene family	autophagy	[48]
ENSSSCG00000008466	*COX72AL*	cytochrome c oxidase subunit 7A-related protein, mitochondrial	motility	[49]
ENSSSCG00000039703	*EEPD1*	endonuclease/exonuclease/phosphatase family domain containing 1	maintenance of genome stability	[50]
ENSSSCG00000005454	*TXNDC8*	thioredoxin domain containing 8 (spermatozoa)	protection against oxidative stress	[51]
ENSSSCG00000003913	*MAST2*	microtubule associated serine/threonine kinase 2	cytoskeletal regulation	[52]
ENSSSCG00000003760	*TTLL7*	tubulin tyrosine ligase like 7	cytoskeletal regulation	[53]
ENSSSCG00000023728	*TECTA*	tubulin-specific chaperone cofactor E-like protein	cytoskeletal regulation	[54]
ENSSSCG00000002430	*EFCAB11 (EFCAB2)*	EF-hand calcium binding domain 11	motility	[55]
ENSSSCG00000031940	*GAS2*	growth arrest-specific protein 2	cytoskeletal regulation and apoptosis	[56]
ENSSSCG00000012002	*ROBO2*	roundabout guidance receptor 2	predicted: fertility	[57]
ENSSSCG00000007741	*NUPR2*	nuclear protein 2, transcriptional regulator	uncharacterized	*n/a*
ENSSSCG00000034914	*CD163L1*	CD163 molecule-like 1	immune response	[58]
ENSSSCG00000016671	*ITPRID1*	inositol 1,4,5-trisphosphate receptor (ITPR) interacting domain containing 1	predicted: receptor binding	*n/a*
ENSSSCG00000029281	*LOC100621701*	protocadherin alpha-C2	predicted: spermatogenesis	[59]
ENSSSCG00000007010	*ZMAT4*	zinc finger matrin-type 4	predicted: fertilization	[60]
ENSSSCG00000030539	*KLK15*	kallikrein related peptidase 15	spermatogenesis	[61]

## Data Availability

Raw sequencing data in the fastq format are accessible in the NCBI-SRA database, BioProject: PRJNA415904 (accession number: SRP121647) and BioProject: PRJNA454080 (accession number: SRP143583).

## Supplementary Materials

The following are available online at https://www.mdpi.com/2075-1729/10/11/300/s1, Supplementary Figure S1: Splicing features of long non-coding RNAs (lncRNAs) of boar spermatozoa; Supplementary Table S1: Differentially expressed long non-coding RNAs (DElncRNAs) in boars with good and poor semen freezability; Supplementary Table S2: Potential *cis*- and *trans*-regulatory targets of differentially expressed long non-coding RNAs (DElncRNAs) in boar spermatozoa; Supplementary Table S3: Gene Ontology (GO) classifications of potential protein-coding genes of differentially expressed long non-coding RNAs (DElncRNAs) in boar spermatozoa.
