# Supplementary material for: Regulatory Potential of Long Non-Coding RNAs (lncRNAs) in Boar Spermatozoa with Good and Poor Freezability
Source: Life (Basel). 2020 Nov 21;10(11):300. doi: 10.3390/life10110300 (PMC7700223; doi:10.3390/life10110300)
Supplement: Supplementary file 1 [file life-10-00300-s001.zip › life-987176 supp xml/Supplementary Figure S1.pdf]

A

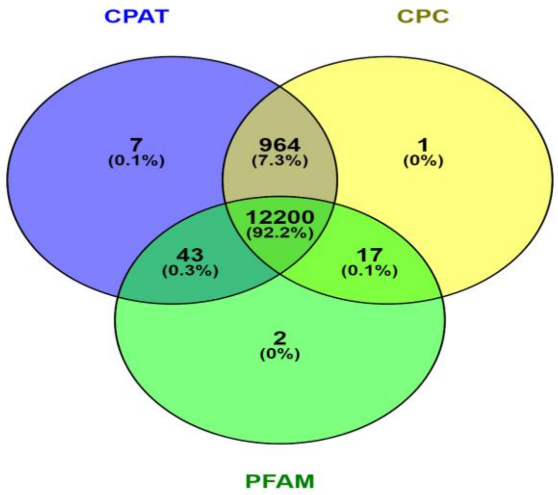

B

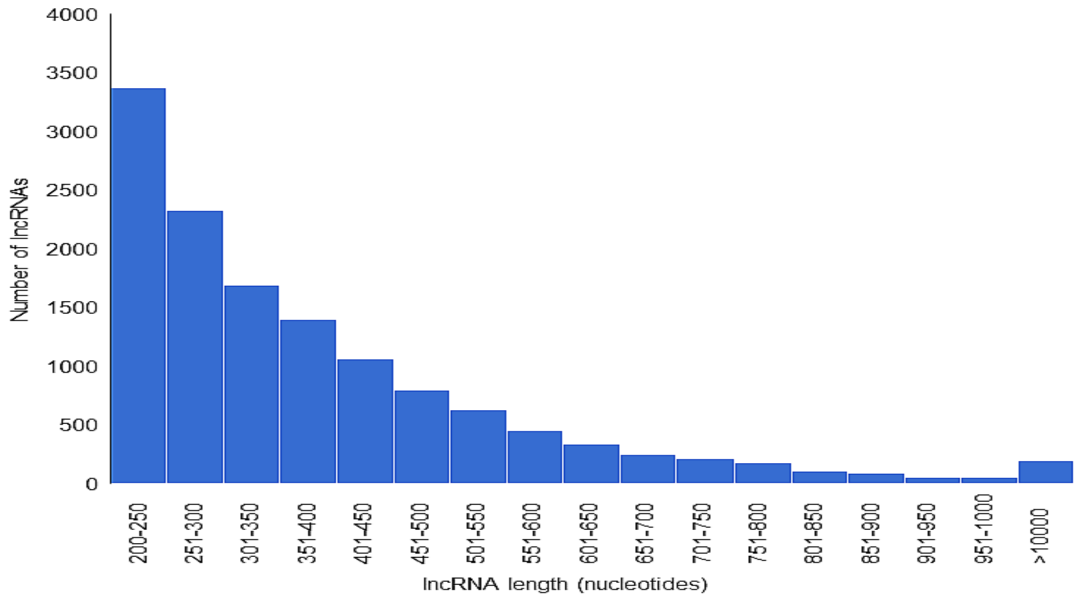

C

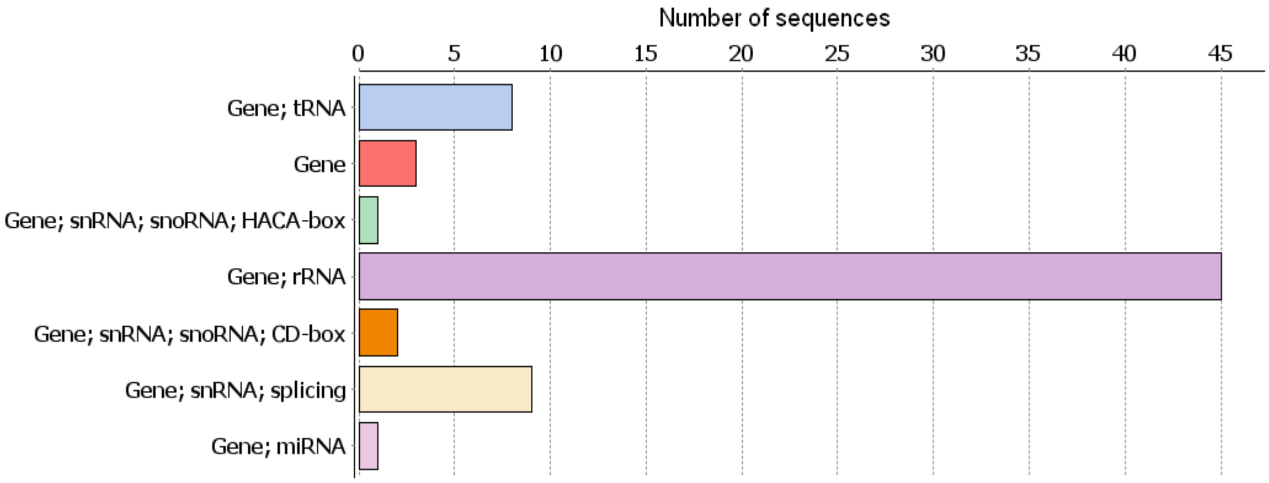

Supplementary Figure S1. Splicing features of long non-coding RNAs (lncRNAs) of boar spermatozoa. (**A**). Venn diagram showing overlapping and non-overlapping splicing isoforms of lncRNAs. (**B**) Distributions of lncRNAs with length for BLAST sequences. (**C**) Rfam distributions of biotype sequences detected in lncRNA splicing isoforms.
